# *Bradyrhizobium denitrificans* strain SoilA*,* isolated from dryland mesquite litter soil in Arizona, USA, grows using calcium oxalate as a sole carbon source

**DOI:** 10.1128/mra.01126-25

**Published:** 2026-01-30

**Authors:** Alexander Sonke, Christine Quan, Elizabeth Trembath-Reichert

**Affiliations:** 1 School of Earth and Space Exploration, Arizona State University 7864, Tempe, Arizona, USA; 2 School of Life Sciences, Arizona State University 7864, Tempe, Arizona, USA; University of Wisconsin-Madison, Madison, Wisconsin, USA

**Keywords:** oxalotrophic, *Bradyrhizobium*, dryland

## Abstract

A bacterial isolate capable of utilizing calcium oxalate as its sole carbon source was isolated from desert soil to explore the role of oxalotrophy in drylands. Complete genomic sequencing and phylogenomic analysis identified the organism as a strain of *Bradyrhizobium denitrificans*.

## ANNOUNCEMENT

We isolated *Bradyrhizobium denitrificans* SoilA from urban desert soil collected beneath an oxalogenic mesquite tree (*Neltuma sp*.; GPS coordinates: 33.418399, −111.902891). A sterile 50-mL Falcon tube was used to scoop the soil, including litter, from below the mesquite tree, on 29 March 2022. The soil was incubated in liquid Schlegel mineral medium (SMM) (1) at 30°C and isolated by serial dilutions in SMM using calcium oxalate as the sole carbon source, followed by transfer to solid SMM agar plates as described in reference 2, and subsequent streak-plating. Growth was observed from 10°C–40°C, with optimum growth at 35°C. Strain SoilA is the first oxalotrophic *Bradyrhizobium* isolated from desert soil—an underexplored metabolism in drylands (3).

DNA extraction, library preparation, and sequencing were performed by SeqCoast Genomics (Portsmouth, NH, USA). Cells were scraped from agar plates with pipette tips and deposited directly into MagMAX Microbiome bead beating tubes. Samples were extracted using the Qiagen DNeasy 96 PowerSoil Pro QIAcube HT Kit (#47021).

DNA samples were prepared for whole genome sequencing without shearing and using the Oxford Nanopore Technologies Native Barcoding Kit (#SQK-NBD114). Long fragment buffer was used to promote longer read lengths. Sequencing was performed on the PromethION 2 Solo platform using a FLO-PRO114M Flow Cell (R10 version). Sequencing was performed with a translocation speed of 400 bp. Base calling was performed on the GridION using the super-accurate base calling model with barcode trimming enabled. MinKNOW version: 24.06.10. Dorado version: 7.4.12. 183,144 reads with Q scores of 10 or greater were used for downstream analysis.

Samples were prepared for whole genome sequencing using the Illumina DNA Prep tagmentation kit (#20060059) with Illumina Unique Dual Indexes. Sequencing was performed on the Illumina NextSeq2000 platform using a 300-cycle flow cell kit to produce 2 × 150 bp paired reads. Then, 1%–2% PhiX control was spiked into the run to support optimal base calling. Read demultiplexing, read trimming, and run analytics were performed using DRAGEN v4.2.7, an on-board analysis software on the NextSeq2000.

**Fig 1:**
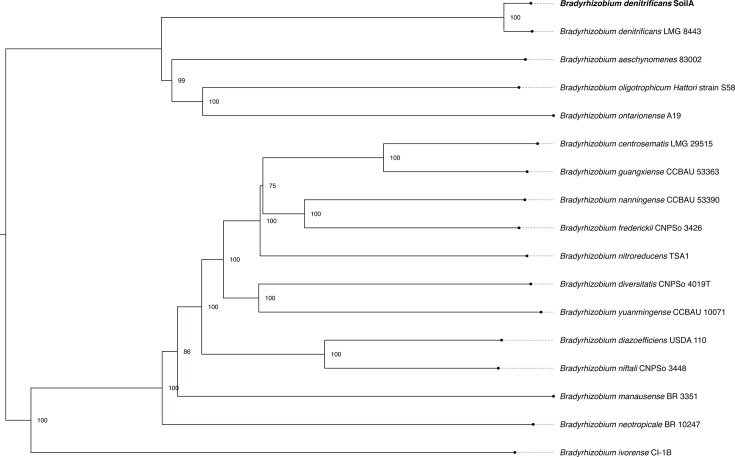
Phylogenomic tree of genus *Bradyrhizobium* genomes and our SoilA isolate (bolded). The tree was created by exporting single-copy genes from the pangenome database (19), removing gap characters >50% of sequences with trimAl (20), then generated with IQ-TREE v2.3.0 (21). Node values indicate bootstrap values >51%.

Genome assembly was performed based on the workflow from Wick et al. (4). Briefly, fastp v0.20.0-gcc-12.1.0 was run on Illumina short reads to remove adaptors and trim off low-quality bases (5). Long-read assembly, circularization, trimming of overlapping ends, and rotation to the dnaA gene were performed using Trycycler v.0.5.5 (6), Flye v2.9.5 (7), bwa-0.7.17-gcc-12.1.0 (8), and a script combining Minimap2 v2.28 (9), Miniasm v0.3/Minipolish v0.1.3 (10). The assembly was polished with Medaka v2.0.1 (2018, Oxford Nanopore Technologies Ltd.) and Polypolish v0.6.0 (11). Analysis was completed using the Sol Supercomputer at Arizona State University (12). Genome statistics are reported in Table 1. Completeness and contamination were checked with CheckM v1.1.6 (13). Genome annotation was performed using the NCBI Prokaryotic Genome Annotation Pipeline (PGAP) v6.10 (14–16). 16S and 23S rRNA sequences were identified in the annotated genome for database submission. The GTDB taxonomy assignment was genus *Bradyrhizobium* with 99.08% similarity to strain *B. denitrificans* LMG8443 (17, GTDB-Tk v.2.5.2 [18]; see Fig. 1 for phylogram).

**TABLE 1:** Genome statistics for *Bradyrhizobium denitrificans* SoilA

Parameter	Result
Genome coverage, Nanopore	~150×
Genome coverage, Illumina	~23×
Completeness, final assembly	99.99%
Contamination	0.91%
Total length (bp)	8,629,737
No. of contigs	1
GC content	64.7%
N_50_	8,399,321
No. of protein-coding genes	7,931
No. of rRNA genes	6
No. of tRNA genes	62
No. of CRISPRs	0
Average nucleotide identity	99.08% ANI with type strain, *Bradyrhizobium denitrificans* LMG8443

## Data Availability

Genomic data are available in NCBI, the DNA Data Bank of Japan (DDBJ), and the European Nucleotide Archive (ENA) under BioSample accession number SAMN50479368 and BioProject accession number PRJNA1302448. Raw sequence reads are available in NCBI under SRA no. SRS26770936. The 16S rRNA gene sequence and complete genome have been deposited in GenBank under no. PX093052 and JBQGLH000000000, respectively. The strain is held by the U.S. Department of Agriculture ARS Culture Collection under the number NRRL B-65762.
